# From Home to Heaven: The Spatial Imaginaries of Nonprofit Organizations

**DOI:** 10.1007/s11266-023-00603-w

**Published:** 2023-09-15

**Authors:** Dominik Karner, Florentine Maier, Michael Meyer

**Affiliations:** Department of Management, Institute for Nonprofit Management, WU Vienna University of Economics and Business, Welthandelsplatz 1, 1020 Vienna, Austria

**Keywords:** Nonprofit organizations, Spatial imaginaries, Organizational space, World polity, World society

## Abstract

We investigate how nonprofit organizations (NPOs) construct imaginaries of their premises, their local environment, and beyond. Based on a qualitative analysis of the websites of 209 randomly sampled NPOs in a metropolitan region in Central Europe, we find four distinct spatial imaginaries: (1) The world polity imaginary constructs NPOs as a part of a spatial environment that is neatly divided into nation states, supranational structures, and subnational units. (2) In the world society imaginary, NPOs are active in blurred, fluid, and overlapping spaces such as networks, commercialized spaces, or natural habitats. (3) In a religious imaginary, the material world is complemented by a transcendental realm and categorized into spaces of the sacred and the evil. (4) Finally, in a lococentric imaginary, NPOs construct a dichotomy between "home" and the alien rest of the world. Each of these spatial imaginaries conveys distinctive ways of situating the organization in their spatial environment and implies specific organizational practices and emotional enchantments of space.

**Supplementary Information:**

The online version contains supplementary material available at 10.1007/s11266-023-00603-w.

## Introduction

Concepts of space have a significant impact on human life. They reflect power structures, technological, and cultural developments and guide collective and individual action. However, as societies change, even concepts taken for granted begin to crumble (Harvey, 1990). Cities, once imagined as imperial capitals, are now conceived as “smart”, “global,” “green,” or “resilient” (Huyssen, 2008). At the global level, the notion of a world governed by autonomous nation states and the vision of an ongoing process of cultural and economic globalization (see, e.g., Meyer, 1987) have come under increasing pressure as a result of terrorist attacks, economic crises, and the rise of nationalism and religious fundamentalism (Norris & Inglehart, 2018). Furthermore, climate change and pandemics have fueled notions of co-dependence of all beings on earth (Latour, 2018). These powerful visions of space—so-called *spatial imaginaries* (Massey, 1999)—permeate societies and shape peoples’ everyday lives.

Nonprofit organizations (NPOs) play central and ambivalent roles in the construction of spatial imaginaries—from industry associations promoting economic globalization (Zhao et al., 2016) to social movement organizations fighting for a just alter-globalization (see, e.g., Katz, 2006). However, NPOs also promote anti-globalist and non-secular imaginaries (Weber, 2013). As important actors in modern societies, NPOs’ visions of space underpin developments such as political protest (Boudreau, 2007), globalization (Massey, 1999), migration (Watkins, 2015), climate policies (Levy & Spicer, 2013; Vandepitte et al., 2019), or urban development (Golubchikov, 2010). Thinking about spatial imaginaries therefore provides an eminent entry point for understanding the diverse roles of NPOs in major societal developments.

Despite the influence of NPOs on spaces at all scales, research on their role in the construction of spatial imaginaries is scarce. The aim of this study is to shed light on this issue by asking: “How do NPOs construct spatial imaginaries of their premises, their surroundings, and larger spaces?” Methodologically, we approach this question by analyzing the websites of a representative sample of NPOs from the Vienna metropolitan region. We apply a Lefebvrian framework to characterize the spatial practices of NPOs (Dale & Burrell, 2008; Lefebvre, 1991).

Looking at NPOs’ spatial imaginaries is particularly interesting for two reasons. First, the impact of organizations on space is pervasive in contemporary society. They are major users and exploiters of spatial resources, and at the same time, they are pivotal for the protection of social and natural spaces. Our focus on NPOs is therefore an instance of looking at the spatial effects of organizations in general. Second, NPOs provide insights into a wide variety of spatial imaginaries. As "hybrids," they operate between markets, governments, and communities, thus spanning different institutional fields (Skelcher & Smith, 2015). This hybridity allows us to analyze a wide range of spatial imaginaries and, through direct comparison, determine their distinctive characteristics.

In the next chapter, we will introduce our key theoretical concept of spatial imaginaries. We will discuss how they are relevant for organizations and summarize how previous studies have engaged with NPOs. After describing our methodology, we present our findings, the four distinct spatial imaginaries. Finally, we discuss the theoretical and practical implications, limitations, and perspectives for further research.

## Spatial Imaginaries and NPOs

Concepts of space determine how actors approach the world. They allow individuals to navigate their physical environment (Tversky, 1991) and to narrate about space (Linde & Labov, 1975). At the same time, cognitive representations of space are continuously updated by actor’s engagement with their surroundings (Piaget, 2013, orig. 1954). Societal fields, such as the economy, civil society, and politics, are of central importance in this context. They shape culturally embedded beliefs and are reproduced in discursive and visual representations of space, thereby serving as templates for individual and collective action (De Certeau, 1988).

In the geographic literature, these “collectively shared worlds of thoughts” (Boudreau, 2007: 2596) have been described with the concept of *spatial imaginaries* (see, e.g., Huyssen, 2008). They represent deeply held beliefs about space, for example, about cultural boundaries or the relationships between different spaces and places (Waters & Barnett, 2018). Thus, spatial imaginaries are much more than static representations of the physical environment. They are embodied spatial discourses brought into existence through collective cognitive, emotional and material practices (Watkins, 2015).

Organizations play a major role in the construction of spatial imaginaries. They nurture imaginaries of their internal and external spaces, which help them to make sense of their manifold socio-spatial relationships (Berti et al., 2018). In doing so, they define their own place in relation to their environment, as well as the roles and positions of actors and physical matter within their internal organizational spaces.

Organizations construct imaginaries of their inner-organizational spaces to influence how actors perceive and act in them (Yanow, 1998). They accomplish this by demarcating and connecting physical spaces (e.g., offices and sales spaces) and creating an order of spaces (e.g., in office buildings or manufacturing lines, Boje & Baskin, 2011). Drawing material and immaterial boundaries between spaces often goes hand in hand with the definition of roles associated with a person's status in/for the organization (e.g., Siebert et al., 2016). These boundaries are also fundamental to delineate the organization from the outside world, which can be important for solidarity, social exchange, and entrepreneurial spirit (Kokkinidis, 2015). Organizations thereby contrast constructs of their own spaces with imaginaries of their environment (Stephenson et al., 2020).

Thus, the construction of a world beyond their premises is another central function of organizations’ spatial imaginaries (Zukin, 2011). Research has shown how organizations base strategic decisions on imaginaries of countries and regions (Vaara & Tienari, 2010), adjust their organizational structures to urban imaginaries of risk and vulnerability (Wissman-Weber & Levy, 2018), or align their practices with local norms to gain and maintain legitimacy (Marquis & Battilana, 2009). Other studies have focused on how organizations construct imaginaries of spaces in ways that increase their market value. Place branding is an essential technique for this purpose (de San Eugenio Vela, 2013). Product branding and the creation of geographically marked product categories (e.g., Australian wine, Croidieu et al., 2016) are other techniques that contribute to place branding.

In human geography, this commodification and colonization of space has been of central interest (Kipfer, 2007; Lefebvre, 1991, 2014). In particular, studies have focused on how capitalism and globalization marginalize local readings of space (Grossi & Pianezzi, 2017). However, as even powerful imaginaries remain simplistic readings of space, scholars have emphasized how alternative imaginaries offer possible avenues of contestation (Healey, 2002). Such tensions have been documented, for example, in conflicts about land use (Ravindran, 2019), migration (Titley, 2019), tourism (Kothari & Arnall, 2017), and climate action (Levy & Spicer, 2013).

NPOs tend to play diverse and ambivalent roles in such spatial discourses. They often challenge hegemonic spatial imaginaries promoted by governments and international corporations by deliberately constructing alternative imaginaries of space (Ravindran, 2019; Wolford, 2004), for example, in opposition to urban development projects (Hincks et al., 2017). In addition, some NPOs create and maintain spaces that shield their users from the outside world, allowing them to live and express unconventional identities and lifestyles – so-called heterotopias (Foucault, 1984; Gallan, 2015; Steyaert, 2010). However, NPOs may also act just like corporations and governments. Studies on urban imaginaries such as “world cities” (Golubchikov, 2010), “smart cities”(Burns & Andrucki, 2021), and “creative cities” (Catungal & Leslie, 2009) suggest their role as catalysts of these neoliberal urban visions. On a larger scale, NPOs promote ambiguous spatial imaginaries related to geopolitics and international development (Bürkner & Scott, 2019).

Despite their diversity, the spatial imaginaries of NPOs found in these studies are largely consistent with central tenets of world polity theory (Meyer, 1987), which describes and explains the spread of “*world culture*” (Boli et al., 2011). NPOs are central actors in world polity theory, promoting universal principles such as human rights and scientific rationality in a globalized world (Meyer & Jepperson, 2000). In addition, NPOs give voice to non-state actors, claim universal de-territorialized rights, and “propagate authoritative rules and standards that states are expected to follow and against which they are judged” (Cole, 2017: 96). From the perspective of this theory, NPOs should construct spatial imaginaries that are consistent with these ascribed actor roles.

However, scholars have criticized NPO research for being biased toward organizations with a liberal and democratic value base, thus systematically ignoring parts of civil society that uphold values that are often normatively described as "uncivil" (Kopecký & Mudde, 2003). Moreover, the focus of world polity theory on global civil society marginalizes conceptions of space that are rather disconnected from global structures (Munck, 2002). Developments, such as the rise of right-wing populism, religious fundamentalism, and economic protectionism, have further fueled this skepticism (e.g., Norris & Inglehart, 2018). Based on this critique, we expect that NPOs may construct a wide variety of spatial imaginaries, some of which differ from “world culture”.

## Methods and Empirical Setting

### Setting, Sampling, and Preparing the Data

We address our research question by examining a representative sample of NPOs [applying the definition by Salamon and Sokolowski (2016), but excluding foundations] in the Vienna metropolitan region. With a population of 2.5 million, this region is one of the largest agglomerations in Central Europe. It serves as a hub for the wider Central European region and is home to organizations of the Austrian federal government, numerous headquarters of national and international companies and NPOs, and several international organizations. In total, there are over 20,000 NPOs in this region. Our findings, therefore, come from a globally interconnected region.

We compiled a list of the total population of NPOs in the region (n = 20,280) from the pertinent registers, namely the company register and the register of associations.^1^ We used register data from 2017. We followed the eligibility criteria established by Salamon and Sokolowski (2016), thus including only self-governing private organizations with limited profit distribution and non-compulsory participation. We excluded purely grant-making foundations (which are of minor relevance in the Austrian third sector anyway). From this list, through random number assignment and sorting, we randomly selected a target population of 369 active NPOs. We compiled contact information and found that more than half had a website (n = 209). The other organizations presented their contact information and rather sparse content on the website of an umbrella association or of the municipality where they are located, had just a Facebook page, or had no web presence at all.^2^

Since we intended to approach websites as sources of rich qualitative data about how NPOs present themselves to the world and how they imagine that world (Powell et al., 2016), we focused on organizations with a website of their own. These organizations are typical for NPOs in the region (see descriptive data in the supplementary material). We downloaded the websites in 2018, converted them to PDF format, and imported them into MAXQDA. Almost all text was in German. Seven websites contained content in English, one in Japanese, one in Russian, and one in Romanian. In the case of the German and English language websites, two of the authors coded each website with the third author as a sparring partner to check emerging codes and interpretations for plausibility. For the few websites in other languages, we additionally involved a native speaker or professional translator. Wherever ambiguities or disagreements about interpretations arose, we sought consensus within the team of authors, and translators or native speakers when necessary.

### Analyzing the Data with a Lefebvrian Framework

We analyzed the websites with methods of qualitative text and visual analysis. In the first step, we identified all images or bits of text from the NPOs’ websites that referred to space while also carrying some narrative meaning, thus basically including all references to space except pure address information. We then coded the data applying a scheme based on the Lefebvrian idea of the social production of space (1991), specifically his so-called *spatial triad*. This heuristic describes three *trialectically* related spatial dimensions, the conceived, the perceived, and the lived space (Lefebvre, 1991). Dale and Burrell (2008) adopted Lefebvre’s ideas for the study of organizations, distinguishing three types of organizational practices through which organizations construct spatial reality:

- *Emplacement* organizes space by aligning objects and actors with their rightful places. Organizations emplace by enclosing, partitioning, classifying, and ranking space, yielding a distinct arrangement of places and their appropriate actors and activities (Dale & Burrell, 2008: 53f). We analyzed texts and images with the following question in mind: "In this image or text, where does the NPO locate itself, others, or an issue?" Similar ways of answering this "where" question were grouped into codes for emplacement.
- *Enchantment* charges space with emotions. Organizations achieve this, for example, through multimodal campaigns to brand locations, or by placing symbols in the built environment. We screened the texts and images with an eye on the creation of emotionally moving meanings of space. Similar ways of making space emotionally moving were grouped into codes for enchantment.
- *Enactment* is about the construction of space through interacting in and with space, moving through space and shaping space through embodied practices (Dale & Burrell, 2008: 66). We searched for information about the activities of actors in texts and images. We coded for enactment by focusing on how actors enact space through social practices in the course of the organization's activities. Similar ways of enacting space were grouped into codes.

In coding for emplacement and enchantment, we approached the data with a discourse analytic mindset, interpreting how images and texts produce meanings of space. To code for the enactment, we approached the data with a content analytic mindset, reading the images and texts as transparent sources of information about practices. In the supplementary material, we provide an overview of the coding scheme with typical examples of coded text and images. Finally, as we coded emplacement, enchantment, and enactment, patterns emerged among these codes. Different types of emplacement tend to occur in combination with distinctive forms of enchantment and enactment. These patterns condensed into the four spatial imaginaries.

## Findings: Four Spatial Imaginaries

The four distinct patterns of ordering the world and thus achieving emplacement, accompanied by typical forms of enchantment and enactment of space constitute spatial imaginaries (see Table 1).

**Table 1:** Four spatial imaginaries

	World polity	World society	Religious	Lococentric
Emplacement	At the national level Federal structures above and below the national level	Networks Regions Globally and locally	Transcendent and immanent realm Places of unique spiritual significance Places of worship	Home Foreign parts
Enchantment	Patriotism	Empathy with the biosphere and distant suffering others Marketized attractiveness	The world as God’s creation Evil Sacred	Full of close personal ties Uniqueness of home
Enactment	Building and maintaining federal structures Work in designated spatial domain Standardization, certification Permanent comprehensive competition systems	Fluid mobility Global agency on behalf of others Researching and monitoring global issues Location branding	Prayer Pilgrimage Missionary activities	Social gatherings Touristy travels

Before detailing our findings qualitatively, we provide some quantitative results to put these findings in context: The world polity imaginary is most prevalent; it is mobilized by 67% (n = 140) of the organizations. The world society imaginary follows with 64% (n = 134) of NPOs. The lococentric imaginary is drawn upon by 23% (n = 48) of NPOs and the religious imaginary by 5% (n = 11). Organizations rarely draw on only a single spatial imaginary. Often, they employ two or even more imaginaries in separate parts of their website. In the following section, we describe the four spatial imaginaries. For each imaginary, we also present an ideal–typical vignette of an NPO conveying that imaginary. In addition, we provide visual data to enrich these vignettes, an overview of the practices characteristic of each imaginary, the coding scheme we developed to describe them, and additional descriptive information in the supplementary material.

### The World Polity Imaginary

The world polity imaginary corresponds to the worldview underlying the institutional system described by this label (Cole, 2017; Meyer, 1987). It depicts a federally structured world that is split into mutually exclusive and exhaustive national jurisdictions. Nation states are the key spatial units in this imaginary. They are themselves subdivided into smaller units, such as provinces, and relate to each other through intergovernmental organizations and supranational entities. NPOs mirror this hierarchical order in their structures: At each level of the federal hierarchy, there are specific NPOs with specific functions (e.g., international, European, and national umbrella associations). Thus, this imaginary is typical of federally structured organizations that include local, provincial, and national units. In many cases, they are also linked to European and international organizations.

NPOs *emplace* themselves at a particular level in this hierarchy. Clear-cut political boundaries define the NPOs' scope of action and delineate the central actors and institutions. The national level is the pivotal category. Below the national level, many organizations emplace themselves in provinces, districts and municipalities; (e.g., “*[the NPO] is one of the largest and most important leisure- and environmental organizations in the country [Austria], with 460 municipal and 9 provincial chapters)*. Above the national level, we find emplacements at the level of the European Union and NPOs operating on par within United Nations institutions.

Practices of *enchanting* space in this imaginary were rather unidimensional, namely patriotism at the national level. The NPOs in our sample express pride and commitment to the Austrian nation: “*Austrian [sic]—this is our home country—patria*”. The most explicit and common enchantments of the nation could be found in sports organizations, but also in business associations emphasizing their nationality as a sign of quality by stating “*We are an Austrian organization*.” We could not find comparable enchanting at subnational or supranational levels.

NPOs *enact* the world polity imaginary in four major ways: First, they build and maintain federal structures. This typically involves intensive communication with sister and parent organizations, e.g., “*sending a delegation of six people*” to participate in democratic procedures. Second, NPOs adhere to their designated spatial domain. Often, they take responsibility for a particular problem in that domain, e.g., by helping poor people in a particular Austrian district, or representing interests at a particular spatial level: “*We represent the music industry in Austria*.” Third, NPOs engage in standardization and certification: They negotiate, issue and safeguard standards, and certification schemes that regulate affairs within their spatial domain: “*With the certification of golf schools, the [NPO] has created a tool to ensure the quality of golf instruction in Austria*.” Fourth and finally, competitive arenas are a way of enacting the world polity imaginary. Such arenas exist in sports and other fields of nonprofit activities, from music to animal husbandry. They connect lower spatial levels to higher levels via intermediate levels: “*The three best from Austria take part in the international young beekeepers competition.*”

The Austrian Budgerigar Association is a typical example of an NPO promulgating the world polity imaginary. It is the umbrella organization for Austrian budgerigar breeders, structured federally and encapsulating provincial and local groups. The organization is a member of the World Budgerigar Organization (WBO), which organizes issues of budgerigar breeding worldwide in a structure neatly divided into continents (as symbolized in their logo, see supplementary material). The NPO stresses that establishing such a national structure has been a prerequisite for ensuring comparable, high-quality breeding results, and is proud of having unified the Austria breeders, e.g., by organizing national and provincial budgerigar shows. At these shows, judges evaluate the birds and select winners in various categories. The winners may take part in international competitions. These shows also aim to disseminate international breeding standards as defined by the WBO. The Austrian Budgerigar Association also nurtures contact with sister organizations from other countries.

### The World Society Imaginary

World society refers to the "stateless" dimension of the global institutional system (Meyer et al., 2015). World society denotes a flexible, cosmopolitan, diverse imaginary of space in which non-state entities are key actors. It depicts a borderless world with fluid transitions between the spatial categories that the world polity pattern differentiates so clearly. NPOs promulgating this imaginary tend to deal with issues that do not stop at national borders (e.g., migration, global trade).

NPOs see themselves and their beneficiaries *emplaced* in dynamic and complex spatial categories, such as networks and markets, as well as cultural and biological regions. These spaces are not sharply delineated from the outside, or their boundaries are not congruent with political boundaries, e.g., “*the rainforest*”*,* “*the Third World*”. An important pair of distinct spatial categories is the global and local spheres: NPOs present themselves and their issues in a global context, stressing that actions performed on the local level always affect the global.

Two major ways are used for *enchantment* of space: empathy and marketized attractiveness. Enchantment through empathy often refers to the world as an actor. Expressions such as *"Mother Earth"* or *"our beautiful nature"* are characteristic. The biosphere is understood as powerful, life-giving, but also threatened. Empathic enchantment portrays certain places as humanitarian “*crisis regions*” or as ecologically endangered asking, e.g., “*who is helping the oceans?*” In contrast, enchantment through marketized attractiveness portrays certain places as desirable and inviting to spend time and money. A distinctive way of presenting places as attractive is to construct them as glocalized places, e.g., a “*worldwide unique center of expertise*”*,* or a multicultural place offering “*products from all over the world*”.

The spaces of world society are *enacted* in four major ways: First, space is enacted through fluid mobility. The actors of world society belong to networks that transcend national borders. They may be underprivileged groups, such as refugees and working migrants, or members of global elites who frequently cross national borders by working abroad and traveling to “*international conferences*”*,* “*festivals*” or “*car meetings in the DACH region*”. Second, the world society imaginary is enacted by engaging in global agency on behalf of others, e.g., by providing humanitarian aid in troubled regions or by participating in “*the first Reef parade on World Oceans day*”. Third, another enactment practice is researching or monitoring global issues. For example, NPOs may monitor developments though “*satellite surveillance of a rainforest*” or by transmitting “*images and videos recorded by Ghost Cams to the internet*”*.* Fourth and finally, there is location branding, which applies branding techniques to develop the image of places such as cities and neighborhoods to create marketized enchantment, e.g., through marketing events promoting a particular location, or an “*interactive virtual guide through a shopping street*”*.*

*One Billion Rising* is an ideal typical example for the world society imaginary. This NPO is part of a “global movement” of NPOs that advocate for the end of violence against women and children. They understand the oppression of women as a global problem that takes on various forms in various places. Thus, the nonprofit mentions nation states only to identify local facets of this global problem. One Billion Rising has the vision of “*a world without violence and hatred, without borders and war, where all humans can exist with equal rights in a world worth living.*” They portray the world as imbued with violence. At the same time, they point to a possible solution: worldwide solidarity and tolerance. The NPOs’ practices are in line with their vision. Their most visible practice is the annual celebration of world women’s day. On that day, the NPO organizes dance events in public places throughout Austria, while sister organizations conduct events in other locations, to call for an end to violence against women and children.

### The Religious Imaginary

This imaginary parallels the world society imaginary in that it also constructs a borderless world. Apart from that, however, its emplacements, enchantments, and enactments of space have little in common with the secular world society imaginary. The religious imaginary is based on a dichotomy between the immanent and the transcendent realms. The whole world is God's creation and populated by people who are all at least potential believers. This immanent world, however, is also the place where sin and evil reign. Religious practices and sacred places bring people closer to the divine. Predominantly, this imaginary is conveyed by religious organizations.

The religious imagery offers a binary construction with this world and the otherworld. NPOs *emplace* themselves and others in the transcendent and the immanent realms, places of unique spiritual significance, and in places of worship. The world is God's creation; political boundaries or secular administrative systems are irrelevant. The view on the earthly world is decidedly globalist. It is one world, and all people can become believers and join a global community. The NPOs themselves are not entirely rooted in this immanent world, but are connected a transcendent realm beyond what is visible to the eyes of the unbelievers, e.g., *"the realm of God"*, *"the universe"* [in a spiritual sense]. Another spatial category is places of unique spiritual significance: the “*Kaaba in Makkah*”, the biblical *"Holy Land"*, or “*Rome*” as the seat of the Pontifex. Not quite as unique, but also special, are places of worship. These include places where believers practice their fate, from the private room where someone prays in secret, to relatively plain community centers and lavishly adorned mosques.

The religious imagery relies heavily on the *enchantment* of spaces, emphasizing their divine origin, and normatively marking places as evil or sacred. Feelings of revulsion are evoked by portraying specific regions or even the whole world as “*a sinful place where evil reigns*”*.* Feelings of awe are mobilized by characterizing certain places on earth as *"a piece of heaven"*. These spaces are windows to the transcendental realm and—more than ordinary, profane places—represent transcendence in the immanent world.

*Enactment* of religiously imbued space applies a range of practices: “*Praying*”*,* “*pilgrimage to holy places*,” and engaging in “*missionary activities*”. Praying, in particular, is a versatile practice that has the potential to transform any place into a religiously enacted space: “*Everything we do, we do in honor of God*”.

The NPO *Bethabara* is a typical example. Its very name refers to a place of unique spiritual significance: Bethabara is the place on the banks of the Jordan River where Jesus was baptized, and where the Holy Spirit appeared in the shape of a dove. Both events are symbolized in the NPO’s logo (see supplementary material). The website is full of references to the presence of the transcendent in the immanent world, thereby spreading a sense of enchantment with sanctity. All places mentioned on the website have a religious meaning. The nonprofit’s head office is in Maria Taferl, a Catholic pilgrimage site in Austria where the Virgin Mary is said to have worked miracles. The organization runs an education center where the NPO spreads its religious message. It also organizes regular *"prayer circles"* where members meet in person to practice their faith.

### The Lococentric Imaginary

The lococentric spatial imaginary constructs a space identified as home. The organization rests in a local space that is the home of its members, charged with positive emotions. Websites promulgating this imaginary provide thick descriptions of a lifeworld detached from higher-level spaces. What matters is the directly experienced space: the space of everyday life. Everything beyond that home is a more or less indistinct alien space. Actors in this imaginary know each other well and meet in a family-like atmosphere. This imaginary is typical for NPOs that are deeply embedded in a local context. They often serve members and beneficiaries in their immediate surroundings, such as local sports clubs, social clubs, and small social service providers serving a local community.

*Emplacement* centers on the spatial category of home. Everything else falls into the remainder, classified as foreign, which is a largely undefined alien space. Home describes a place where the organization and its members belong and that belongs to them. It is *"our clubhouse", "our stadium"*, *"our shooting range", "our neighborhood",* or *"hometown".* The foreign, in contrast, is hardly elaborated on. The home seems disconnected from larger structures at the national or global level. If at all, members interact with actors from other places on an ad-hoc basis or based on personal contacts, e.g., with friends from other places that the members have got to know through personal contacts. Foreign places—if mentioned at all—only ensure the organization of its own local identity.

In the lococentric imaginary, *enchantment* emphasizes a deep emotional connection to this particular place, home. Close personal ties among people who create a family-like atmosphere make home so special. The uniqueness of home differs from the way the world society imaginary enchants places as uniquely attractive. Home can boast no superlatives or miracles. On the contrary, the descriptions of home seem unpretentious to outsiders. This place is significant as people's home, and it does not matter what else it represents—that is the basic message, e.g., “*Excellent atmosphere at the open air concert in the garden of the parish house*”*.*

*Enactment* concentrates on social gatherings in a warm, personal, intimate atmosphere, e.g., parties, weddings, casual competitions, or games. These gatherings also include trips beyond the organization's premises, e.g., hiking or day trips within a locally confined area considered the homeland. Even traveling together in the style of staid tourists enacts home. Members may go on group travels to tourist destinations, displaying their local identity: “*After the concert [of the brass band], our tour guide led us through ancient Rome.*” This kind of travel is very different from the busy cosmopolitans in the world society imaginary, and the representatives and delegations connecting different levels in the world polity imaginary. In the lococentric imaginary, travelers always display the emotional attachment to their home.

The *HCV Hobby Center Vienna* (name originally in English) exemplifies this imaginary. This organization has three sections: Table tennis, travels, and *"play and fun."* The table tennis section participates in local competitions. In the part of the website devoted to these competitions, the world polity imaginary prevails. Otherwise, the organization promulgates the lococentric imaginary. The travel section organizes group trips via a local travel agency. The *"play and fun"* section organizes leisure events such as casual card game evenings and parties. The history of the organization and the tedious process of finding its home base fill a large part of the website. The HCV mentions past and present members with gratitude, highlighting their services. The website is full of pictures of homey social gatherings in the organization`s premises. Moreover, it shows parts of these premises that are particularly relevant, such as showers, the table tennis area, and most importantly, the bar, which is referred to as *"the center of the association."* Members celebrate parties and watch sports events together. They also emphasize that they have a smoking area. In sum, the website conveys an image of a homey, historically evolved microcosm decoupled from the outside world.

## Discussion and Conclusion

The aim of our study was to analyze the spatial imaginaries constructed by a wide range of NPOs. We identify four spatial imaginaries: A world polity imaginary depicting a federally structured world neatly divided into nation states; a world society imaginary based on networks, regions, and coinciding global and local levels; a religious imaginary based on a binary construction between the transcendent and the immanent; and the lococentric imaginary with home as the central spatial category.

Applying a Lefebvrian perspective, we show how NPOs align practices of emplacement, enchantment, and enactment (Dale & Burrell, 2008) to form these embodied spatial discourses (Watkins, 2015) that guide and justify individual and organizational action (De Certeau, 1988). Our findings thus allow us to draw three theoretical conclusions:

First, we find that a considerable number of NPOs construct non-secular and identitarian visions of space and therefore do not follow the rational actor roles suggested by world polity theory (Meyer & Jepperson, 2000). Such visions of space have received recent scholarly attention because of their central role in developments such as alter-globalization and the rise of parochial nationalism (Eribon, 2013; Hochschild, 2016; Norris & Inglehart, 2018). The lococentric and religious imaginaries can be read in this way. They draw relatively simple mental maps in contrast to the rather complex, impersonal, and abstract imaginaries of world polity and world society. In doing so, they provide their stakeholders with orientation in a complex world (Ryan et al., 2016). They center around focal places—such as a unique home or sacred spaces—that exist outside the usual social order and thus provide a form of shelter. In Foucauldian terms, these spaces could be described as heterotopias (Foucault, 1984). Consequently, these imaginaries rely on strong enchantments of space, and practices aimed at creating mutuality, common identity, and solidarity. Most likely, these imaginaries strongly appeal to those that resist the imaginaries of a globalized world culture (Boli & Thomas, 1999).

Second, we contribute to the literature on the role of civil society in spatial discourses. Literature has given much weight to imaginaries of urban development (Burns & Andrucki, 2021; Golubchikov, 2010) and anti-capitalist imaginaries (Boudreau, 2007; Ravindran, 2019), in which NPOs tend to have unambiguous roles. Our findings, however, highlight the ambivalence of NPOs in the social construction of space, which—as we argue—reflects the hybridity of the nonprofit sector in general (Brandsen et al., 2005; Skelcher & Smith, 2015). NPOs construct a wide range of imaginaries, but draw largely on the world polity imaginary, which is fundamental to the public sector, and the world society imaginary, which is important to business. More importantly, however, we find that NPOs add diversity to these spatial imaginaries. Specifically, we find sustainability and solidarity-oriented versions of world society alongside a vision of global markets and marketized places. The world society imaginary thus accommodates a wide range of NPOs: From industry associations to environmental groups, both of which enchant spaces as unique superlatives. The world society imaginary also accommodates right-wing chauvinist and feminist groups, both traveling to international meetings and forming transnational networks in similar ways. We also find strong notions of solidarity and reciprocity in the lococentric and religious spatial imaginaries. These imaginaries may seem narrow, but they fulfill important functions in the everyday lives of NPOs’ stakeholders. They offer guidance in environments that are characterized by non-universal norms and reciprocal relationships, e.g., for NPOs providing elderly care in small communities. Therefore, we must not misinterpret and reduce these imaginaries to patterns of reactionary and bigoted groups only.

Third and finally, we add to the literature on organizational space, particularly to the stream focusing on the narrative construction of organizational spaces (Boje & Baskin, 2011; de Vaujany & Vaast, 2013; Ropo & Höykinpuro, 2017). This stream of literature has largely ignored the role of organizations in constructing larger societal spaces. Our study expands the lens, showing how organizations align their imaginaries with consistent patterns of spatial practices beyond organizational boundaries.

We have to consider some limitations when interpreting our findings. First, although we base our analysis on a random sample of NPOs, some kinds of organizations that may also be considered parts of the nonprofit sector are not included in our sample. In our case, this means foundations and organizations incorporated by public law (such as parishes of the Catholic Church and volunteer fire brigades in Austria). Nevertheless, we assume that also these organizations deploy some of the four spatial imaginaries identified. Second, we have attempted to provide thick descriptions of the various spatial imaginaries, but we cannot make robust claims about the impact of these imaginaries. We have provided descriptive information about the number of organizations conveying particular imaginaries, but we have not considered their societal relevance. Finally, and most importantly, what we learn about spatial imaginaries through NPOs' websites is likely only the tip of the iceberg, and ethnographic research may be needed to dive deeper beneath the waterline.

In our analysis, we encountered many interesting questions for further research. First, within the scope of this study, we were not able to delve deeply into frictions between spatial imaginaries. Investigating such frictions would require different research designs, such as examining how organizations struggle with different spatial imaginaries when it comes to specific decisions. Second, it would be interesting to develop a better understanding of the less researched and rarer religious and lococentric spatial imaginaries, and to examine how they conflict, coexist, or synergize with more widespread imaginaries. Third, in our data, we found no evidence of how the spatial imaginary of world polity envisions organizations' internal spaces. This seems odd, especially given that all other imaginaries include organizational interiors, and that the world polity imaginary is also poor in terms of emotional enchantment. Further research could explore how organizations that are strongly committed to world polity construct their internal spaces and what that implies for world polity`s appeal in today's organizational society. Using ethnographic methods might be particularly illuminating for any of these research ideas.

Finally, our analysis has some practical implications. NPOs’ spatial imaginaries need to be consistent with their other activities and most likely shape these activities. When organizations want to reinforce a particular worldview, they do so—deliberately or not—through enactments and enchantments of space. As we have shown, the spatial imaginaries imply strange contradictions, e.g., many environmentalist organizations enchanting and enacting space in basically the same way like the associations of industrial enterprises whose negative impacts they try to combat. We encourage practitioners to reflect on how their organization conceptualizes space. It might be worth considering whether current spatial imaginaries are really aligned with their strategy and culture, or whether elements from alternative imaginaries should be mobilized (e.g., an environmentalism native to places of intimate personal significance). Within most organizations’ strategies and cultures, spatial imaginaries are still an unmarked territory.

### Supplementary Information

Below is the link to the electronic supplementary material.

Supplementary file1 (DOCX 3899 kb)

## Footnotes

1 Register data was provided by Compass Verlag GmbH.

2 We provide a detailed description of our sample in the supplementary material.
