# Supplementary material for: From Home to Heaven: The Spatial Imaginaries of Nonprofit Organizations
Source: Voluntas. 2023 Sep 15;35(2):386–96. doi: 10.1007/s11266-023-00603-w (PMC11039416; doi:10.1007/s11266-023-00603-w)
Supplement: Supplementary file 1 — Supplementary file1 (DOCX 3899 kb) [file 11266_2023_603_MOESM1_ESM.docx]

Supplementary material

# Population and sample descriptives

We found meaningful references to space on 199 of the 209 websites. In total, there were 2,603 such references to space.

Regarding general descriptive data, we obtained information about the age of all organizations in the target population from public registers. Little other information about NPOs is publicly available in Austria, but for 164 of the organizations we could use data from the Civic Life of Cities survey conducted in 2020/21 (see <http://civiclifeofcities.org/>). Based on this data (see Table below), we see that the organizations in our sample are typical for NPOs in the region.

|  | n | Age | | n survey | Members | | Staff | | Volunteers | | Budget € | |
| --- | --- | --- | --- | --- | --- | --- | --- | --- | --- | --- | --- | --- |
|  |  | med. | Ø |  | med. | Ø | med. | Ø | med. | Ø | med. | Ø |
| Target population | 369 | 17 | 25 | 164 | 50 | 10,248 | 0 | 17 | 6 | 62 | 15,000 | 366,292 |
| Sample | 209 | 17 | 26 | 114 | 61 | 9,691 | 0 | 23 | 7 | 82 | 20,000 | 495,481 |
| Excluded from Sample | 160 | 16.5 | 24 | 50 | 22.5 | 11,516 | 0 | 1 | 5 | 13 | 6,600 | 65,731 |
| Web presence on site of umbrella organization | 48 | 23 | 31 | 23 | 70 | 105 | 0 | 2 | 8 | 21 | 6,600 | 116,587 |
| Web presence on site of municipality | 18 | 27.5 | 29 | 9 | 30 | 63,642 | 0 | 0 | 4 | 7 | 8,000 | 16,567 |
| Only Facebook presence | 11 | 5 | 6 | 0 | na | na | na | na | na | na | na | na |
| No web presence | 76 | 15 | 20 | 15 | 10 | 36 | 0 | 1 | 5 | 7 | 6,500 | 25,447 |
| Website not accessible for technical reasons | 7 | 16 | 28 | 3 | 20 | 28 | 0 | 1 | 7 | 7 | 4,250 | 4,250 |

Target population and sample description (figures for members, staff, volunteers and budget are drawn from the 2020/21 Civic Life of Cities survey, which covered 164 of the 369 organizations from the target population. See <http://civiclifeofcities.org/>)

# Codes and coding examples for the spatial imaginaries

## World polity imaginary

| Practices | Code | Example |
| --- | --- | --- |
| Emplacement | National level | 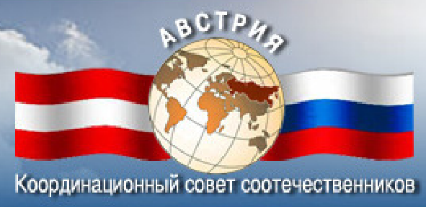 *[The organization's logo consists of a globe with highlighted* ***Russian state territory****, flanked by* ***symmetrically arranged Austrian and Russian flags****.] (o125 – Cultural association)* |
|  | In federal structures above and below national level | “*[The organization is] the* ***umbrella organization*** *of [related organizations]* ***in many European countries****.”* (o319 – environmental organization) |
| Enchantment | Patriotism | *“[The organization] is* ***committed to the sovereign, free and independent Austria and the Austrian nation.*** *We are a* ***patriotic association****. Being Austrian is both an* ***honor*** *and an* ***obligation*** *for us. To avoid any misunderstandings: Patriotism does not equal nationalism! We* ***reject any form of nationalist arrogance****.”* (o33 – catholic student fraternity) |
| Enactment | Building and maintaining federal structures | *“[The organization]* ***has four federations*** *[...]. However, we are already* ***planning to establish an African federation****. All* ***federations are divided into unions****.”* (o219 – service organization) |
|  | Work in a designated spatial domain | *"[The organization] has the mission of* ***meeting the requirements*** *of the VO (EC) 1907/2006 REACH, the RISL (Railway Industry Substance List) and the OECD Recommendation on conflict minerals for SMEs* ***in Austria*** *in an economically sustainable way." (o140 – industry association)* |
|  | Standardization, certification | *“'Fit Sports Austria': […] Please contact the [organization's] coordinator in your province for information about online applications for the* ***'quality seal*** *for healthy exercise and sports in associations'."* (o174 – sports umbrella organization) |
|  | Permanent, comprehensive competition systems | *"Every year, the three best young beekeepers* ***from Vienna*** *are allowed to* ***participate*** *in the* ***national junior beekeepers competition****. […] The three best* ***from Austria*** *then* ***participate*** *in the* ***international young beekeepers competition****"* (o104 – local beekeepers' association) |

*Table 2: Codes for the world polity imaginary*

## World society imaginary

| Practices | Code | Example |
| --- | --- | --- |
| Emplacement | Networks | *"Worldwide networks: Thanks to our professional experience, we can draw on* ***worldwide*** ***networks*** *for the benefit of our clients."* (o386 – nonprofit consultants) |
|  | Regions | *"[The NPO] advocates for a new understanding of the various heterogeneous* ***cultural identities in Europe****, for an open and equal dialogue. With its projects, [the NPO] wants to open up multidimensional approaches, design "****translocal geographies****" […]"* (o205 – arts organization) |
|  | Globally and locally | *"[The NPO] is active at the* ***local*** *as well as at the* ***global*** *level […]"* (o236 – research institute) |
| Enchantment | Empathy with the biosphere and distant suffering others | *"Almost every day we receive social media reports and pictures of our earth that* ***make us feel dread and sadness****."* (o394 – environmental organization) |
|  | Marketized attractiveness | *"The […] festival developed into the* ***unique melting pot*** *that still makes up its* ***special atmosphere*** *today: Interested* ***visitors and international dance stars meet*** *not only in the theaters, but* ***also in the studios, the workshops and the festival parties****".* (o266 – dance festival) |
| Enactment | Fluid mobility | *“When the deceased is received from their home, from the hospital or from abroad,* ***we initiate the necessary steps for the transfer of the body****.”* (0203 – burial society for migrants) |
|  | Global agency on behalf of others | *“[The NPO]* ***helps to build an ecological village*** *in the earthquake-ravaged l'Aquila.”* (o396 – socially engaged soccer association) |
|  | Researching and monitoring global issues | *"For months, animal rights activists were on the trail of calf transports.* ***With extensive photo and video material, they documented*** *the painful journey of dairy calves from Vorarlberg's farms to the giant slaughterhouses in Italy."* (o353 – animal rights organization) |
|  | Location branding | 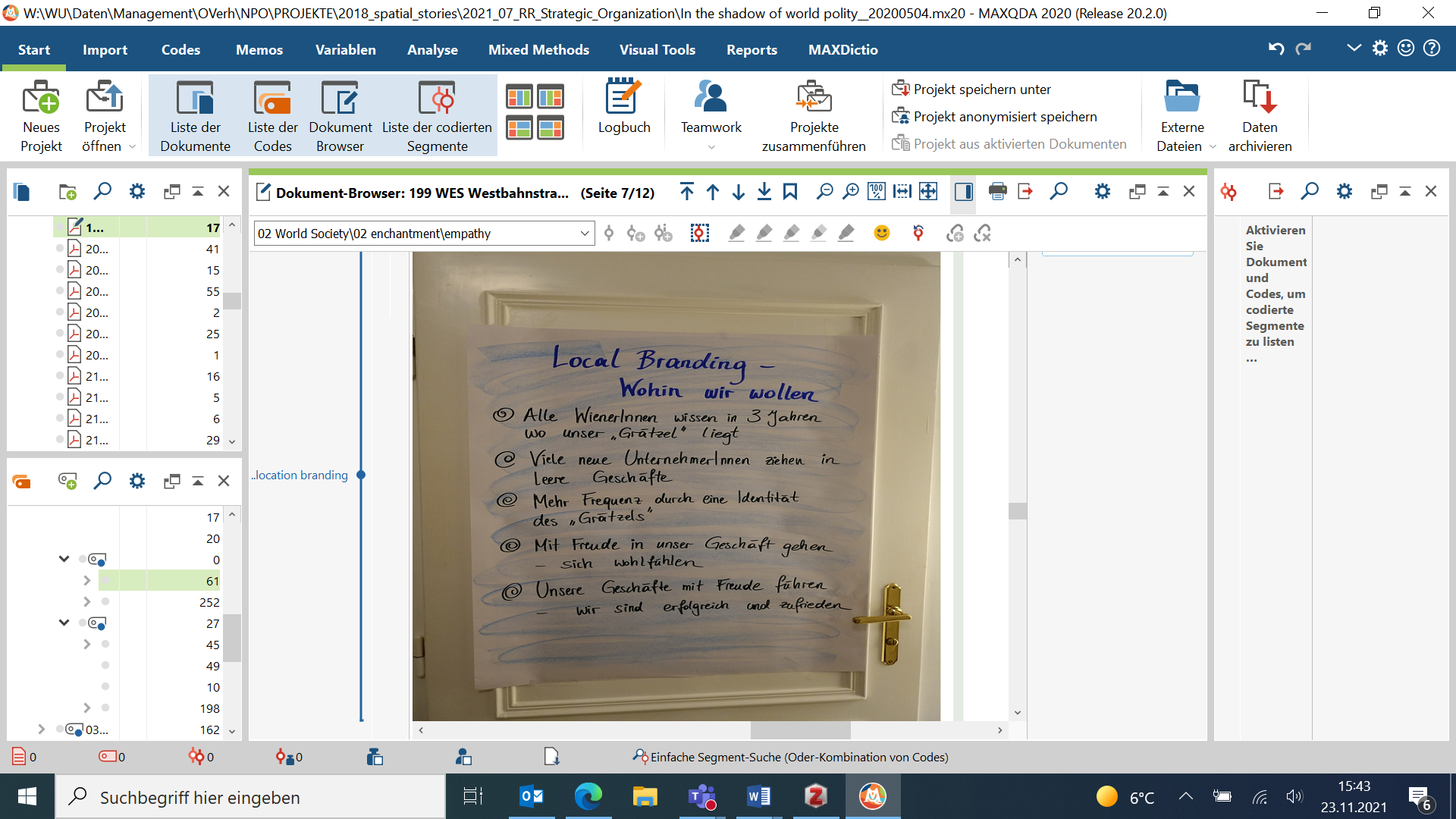 *[Flipchart from a* ***location branding workshop*** *organized by the organization, delineating strategic goals for branding a shopping street]* (o199 – business association) |

*Table 3: Codes for the world society imaginary*

## Religious imaginary

| Practices | Code | Example |
| --- | --- | --- |
| *Emplacement* | Transcendent and immanent realm | *"What faith and love can do: A piece of* ***heaven****. Little miracles. More happiness in* ***the world****."* (o46 – Christian charity) |
|  | Places of unique spiritual significance | *“The* ***sacred mosque in Mekkah*** *represents the center of all Muslims, because the Kaabah, which indicates the direction of prayer, is located in its inner courtyard.”* (o179 – Muslim association and mosque) |
|  | Places of worship | *“The* ***mosque*** *is a place of worship and prayer for Muslims, but also a meeting place for the community”* (o179 – Muslim association and mosque) |
| *Enchantment* | The world as God’s creation | *"The Canticle of the Sun of St. Francis of Assisi is a prayer – more than a cheerfully sung song. It inspires gratitude to* ***God****, who* ***has entrusted us with the earth, his amazing creation****."* (o80 – Christian charity) |
|  | Evil | “*While* ***the world celebrates ‘Halloween’ and the darkness****, we want to be the light*.” (o265 – Christian charity) |
|  | Sacred | *"The purpose of the order was to protect pilgrims in the* ***Holy Land****."* (o100 – Christian military order) |
| *Enactment* | Prayer | “Go into the chamber when you **pray**, close the door, **pray in secret**.” (o5 – Christian association) |
|  | Pilgrimage | *"The first program of this year's* ***Hajj trip, which is organized*** *by the [organization], will take place from 14.08.-30.08.2018 and will cost 3800 €-."* (o179 – Muslim association and mosque) |
|  | Missionary activities | 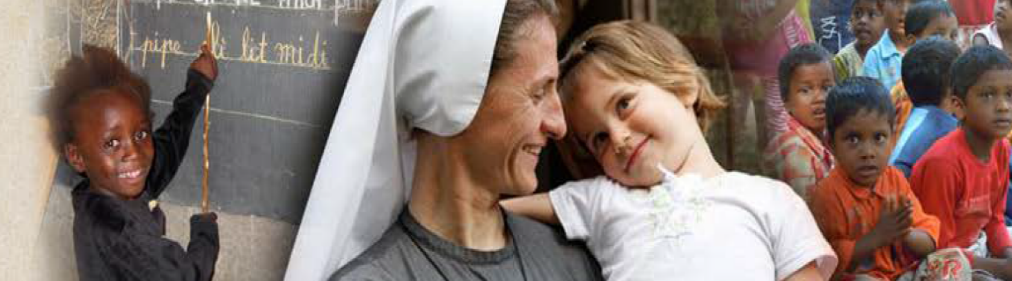  [The banner of the website shows imagery of **Christian missionary work**: A **white convent sister** is **holding** a **white child**; both are smiling. To the left and right are images of **black children in educational settings**.] (o80 – Christian charity) |

*Table 4: Codes for the religious imaginary*

## Lococentric imaginary

| Practices | Code | Example |
| --- | --- | --- |
| Emplacement | Home | *“In* ***our fraternity house*** *there is always something going on.” (o134 – fraternity)* |
|  | Foreign parts | *“On Sunday after breakfast and* ***a short city tour through Feldbach*** *we went back to our home town.”* (o38 – brass band) |
| Enchantment | Full of close personal ties | *“We use the* ***closely knit network of relationships in Kaisermühlen*** *as the basis for our support and care* *work.”* (o184 – nursing home and hospice) |
|  | Uniqueness of home | *“It is about* ***our home[land]****! It is about* **our environment**, quality of life, **our children***!” (o171 – environmentalist citizens` initiative)* |
| Enactment | Social gatherings | *“After a lengthy discussion, the Annual General Meeting was concluded with music and a* ***cozy get-together****.”* (o223 – settlers' association) |
|  | Touristy travels | 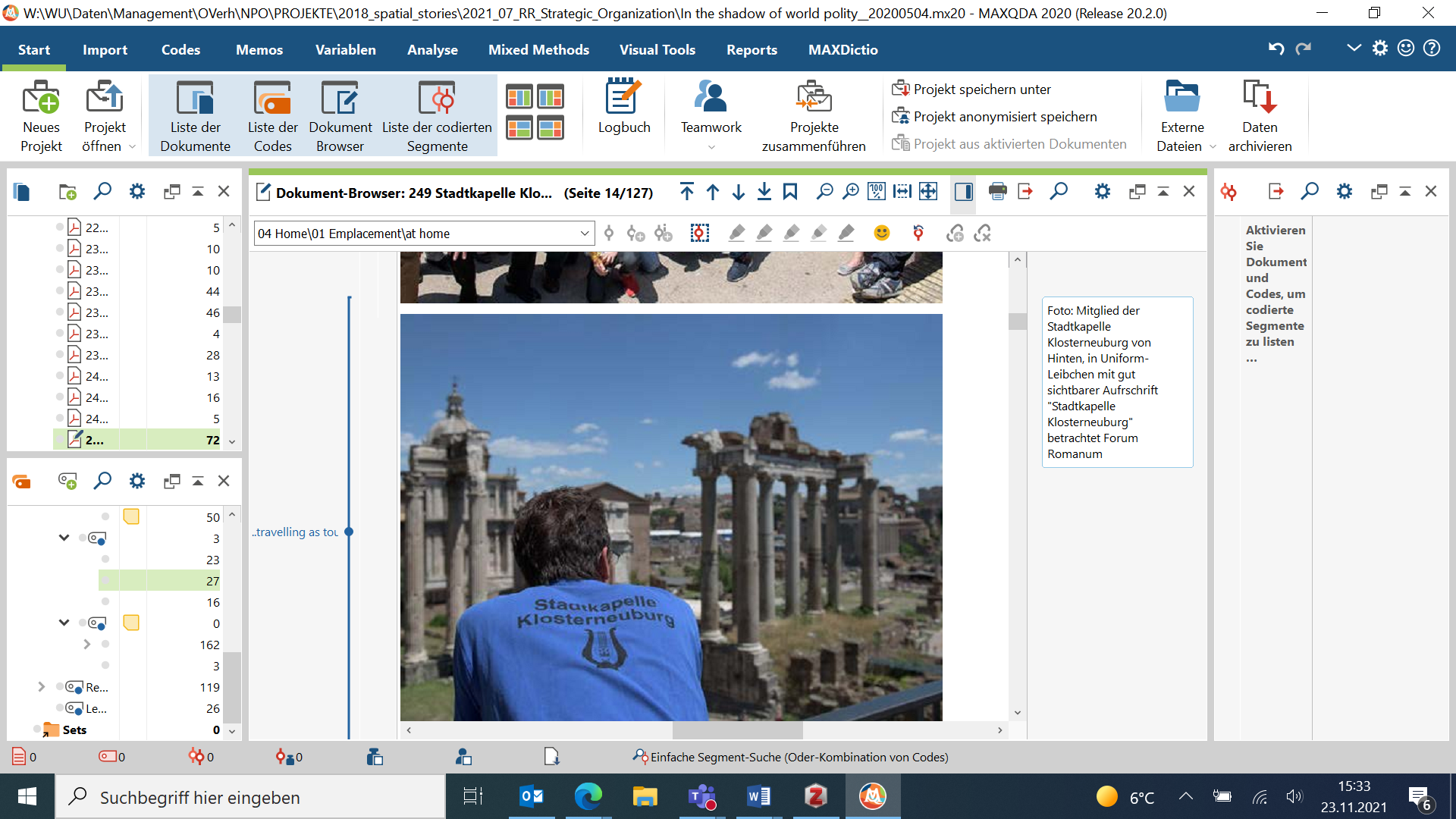  *[Foto: A member of the band,* ***wearing a uniform shirt with the German band name****, is* ***looking at the Forum Romanum in Rome****.] (*o249 – brass band) |

*Table 6: Codes for the lococentric imaginary*

# Visual data of vignettes

## World polity imaginary: Austrian Budgerigar Association


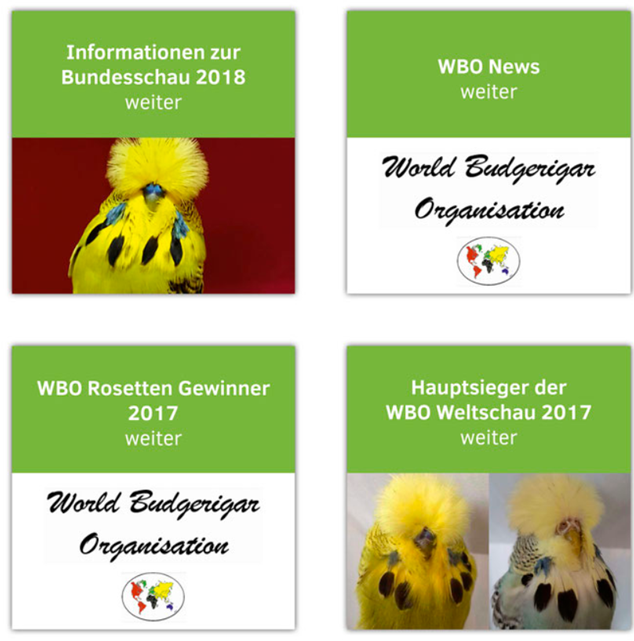


Figure 1: Extract from the website of the Austrian Budgerigar Association (Translation: “Information on the federal show 2018 – continue”, “WBO News – continue: World Budgerigar Organization”, “WBO award winners 2017 – continue: World Budgerigar Organization”, “Champion of the WBO world show 2017 – continue”)

## World society imaginary: One Billion Rising


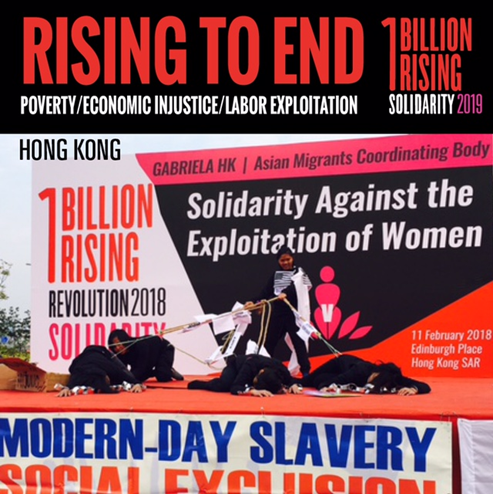


Figure 2: Extract from the website of One Billion Rising

##
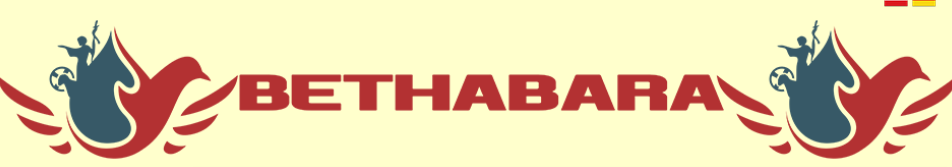
Religious imaginary: Bethabara

Figure 3: Extract from the website of Bethabara, showing the organization’s logo

## Lococentric imaginary: HCV Hobby Club Vienna


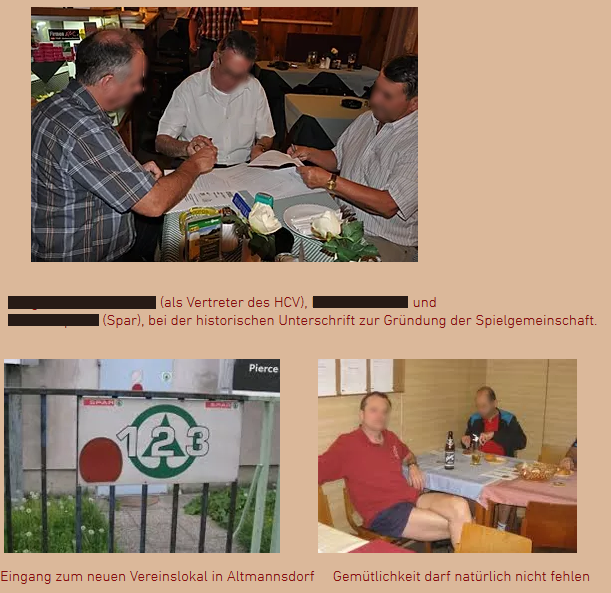


Figure 4: Extract from the website of HCV Hobby Club Vienna (Translation: “[Name of founder] (as representative of HCV) and [Name of landlord] (Spar [an Austrian supermarket chain]) at the historical signature for founding the player’s community; entry to the new clubhouse in Altmannsdorf [a neighborhood in Vienna]; Homeliness is a must”; faces and personal names have been anonymized)
